# Anthrax Toxin-Expressing *Bacillus cereus* Isolated from an Anthrax-Like Eschar

**DOI:** 10.1371/journal.pone.0156987

**Published:** 2016-06-03

**Authors:** Chung K. Marston, Hisham Ibrahim, Philip Lee, George Churchwell, Megan Gumke, Danielle Stanek, Jay E. Gee, Anne E. Boyer, Maribel Gallegos-Candela, John R. Barr, Han Li, Darbi Boulay, Li Cronin, Conrad P. Quinn, Alex R. Hoffmaster

**Affiliations:** 1 National Center for Emerging and Zoonotic Infectious Diseases, Centers for Disease Control and Prevention, Atlanta, GA, United States of America; 2 Villages Regional Hospital, Lady Lake, FL, United States of America; 3 Bureau of Public Health Laboratories, Florida Department of Health, Jacksonville, FL, United States of America; 4 Bureau of Epidemiology, Florida Department of Health, Tallahassee, FL, United States of America; 5 National Center for Environmental Health, Centers for Disease Control and Prevention, Atlanta, GA, United States of America; 6 National Center for Immunization and Respiratory Diseases, Centers for Disease Control and Prevention, Atlanta, GA, United States of America; ContraFect Corporation, UNITED STATES

## Abstract

*Bacillus cereus* isolates have been described harboring *Bacillus anthracis* toxin genes, most notably *B*. *cereus* G9241, and capable of causing severe and fatal pneumonias. This report describes the characterization of a *B*. *cereus* isolate, BcFL2013, associated with a naturally occurring cutaneous lesion resembling an anthrax eschar. Similar to G9241, BcFL2013 is positive for the *B*. *anthracis* pXO1 toxin genes, has a multi-locus sequence type of 78, and a *pagA* sequence type of 9. Whole genome sequencing confirms the similarity to G9241. In addition to the chromosome having an average nucleotide identity of 99.98% when compared to G9241, BcFL2013 harbors three plasmids with varying homology to the G9241 plasmids (pBCXO1, pBC210 and pBFH_1). This is also the first report to include serologic testing of patient specimens associated with this type of *B*. *cereus* infection which resulted in the detection of anthrax lethal factor toxemia, a quantifiable serum antibody response to protective antigen (PA), and lethal toxin neutralization activity.

## Introduction

*Bacillus cereus* infections are typically associated with foodborne illnesses, periodontal diseases, and other opportunistic diseases [1]. However, *B*. *cereus* can be associated with more severe and even fatal infections. Within the last decade, *B*. *cereus* isolates associated with severe infections have been described harboring *B*. *anthracis* toxin genes and/or capsule biosynthesis genes. Hoffmaster et al. described the first *B*. *cereus* with *B*. *anthracis* toxin genes, *B*. *cereus* G9241, to cause a severe pneumonia in a metal worker from Louisiana [2]. Since that report, there have been several additional accounts of infection associated *B*. *cereus* resembling G9241 isolated from severe pneumonia cases in metal workers in Texas [3, 4, 5]. With the exception of the initial case from Louisiana, these infections were fatal. In addition to these isolates, Klee et al. characterized two *Bacillus* isolates harboring *B*. *anthracis* virulence genes, now termed *B*. *cereus* biovar anthracis, cultured from deceased great apes in Cote d’Ivoire and Cameroon. [6].

**Fig 1:**
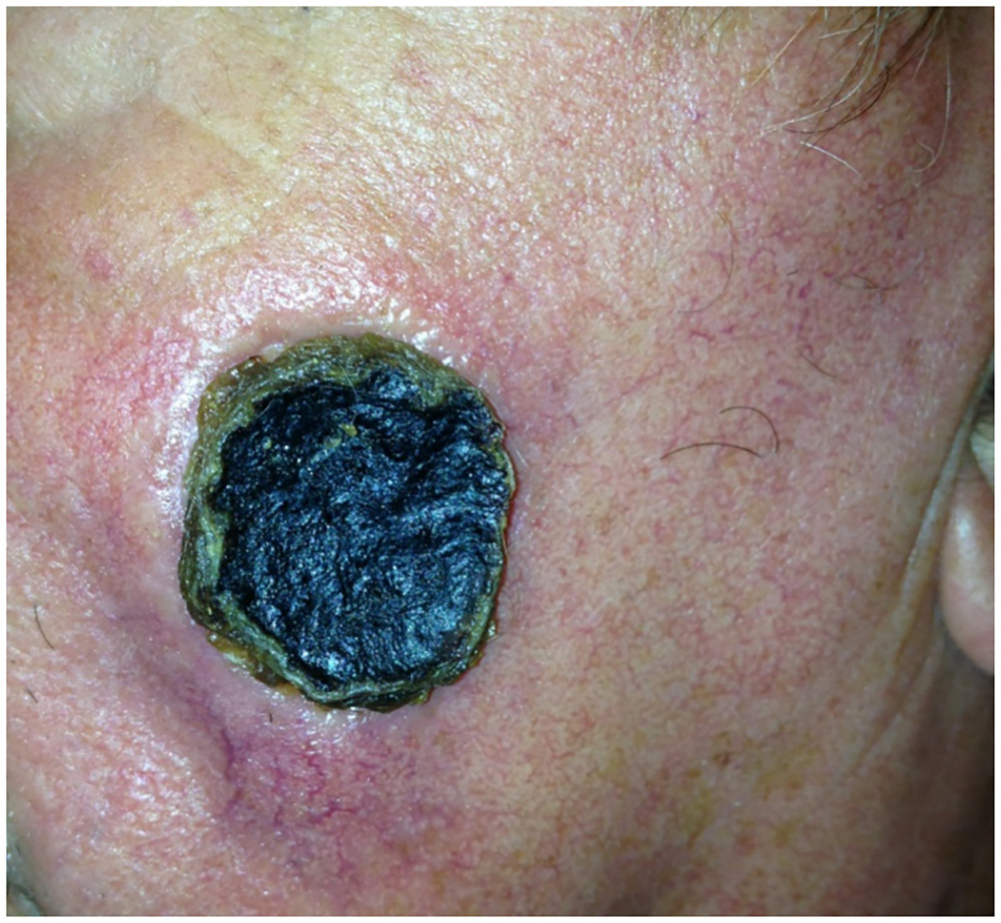
Anthrax-like cutaneous lesion on left check of 70-year-old male Florida resident.

In this report, we characterize a *B*. *cereus* isolate, BcFL2013, cultured from a swab of a facial lesion resembling an anthrax eschar from a 70-year-old Florida resident. (Fig 1). The patient was hospitalized and received antibiotic treatment but fully recovered. The Florida Department of Health Laboratory performed the *B*. *anthracis*-specific polymerase chain reaction assay (LRN PCR) on the isolate from the skin lesion and detected one of the *B*. *anthracis* plasmids, pXO1. The isolate was then forwarded to the Centers for Disease Control and Prevention (CDC) for further characterization. In addition to isolate characterization, plasma and serum samples from the patient were collected and forwarded to the CDC for serological testing.

## Materials and Methods

The BcFL2013 isolate was inoculated on trypticase soy agar with 5% sheep blood (BD Diagnostics Systems, Franklin Lakes, NJ) and tested to determine susceptibility to gamma phage as previously described [7]. Capsule staining and visualization was performed by incubating cells overnight at 37°C in defibrinated horse blood (Remel, Lenexa, KS). Capsules were visualized using India Ink (Remel, Lenexa, KS) under a 100X oil immersion objective. PCR assays for *B*. *anthracis* virulence genes (*pagA*, *lef*, *cya* and *capA)* were performed as previously described [8, 9]. The pBC210 (previously called pBC218) PCR assay was performed as described by Hoffmaster et al. [3].

The isolate was also molecularly characterized by multilocus sequence typing (MLST) and protective antigen gene (*pagA*) sequencing prior to performing whole genome sequencing (WGS). MLST and *pagA* sequence typing was performed as previously described [10, 11]. Briefly, MLST was based on the analysis of seven housekeeping gene partial sequences (*glpF*, *gmk*, *ilvD*, *pta*, *pur*, *pycA*, and *tpi*) and is described online at http://pubmlst.org/bcereus. For WGS, a 101 X 101 paired-end run was performed in an Illumina GAIIx using TruSeq chemistry and yielded 10,182,400 reads. A de novo assembly was performed using CLC Genomics Workbench 6.0.4 (CLC Inc., Aarhus, Denmark) which yielded 84 contigs consisting of 5.46 Mb, with an *N*_50_ of 139,961 bp. [12]. The reads were also mapped against reference sequences for G9241 (CP009590.1) and plasmids associated with G9241, pBC210 (CP009591.1), pBCXO1 (CP009592.1) and pBFH_1 (CP009589.1), to extract homologous sequences from BcFL2013 using CLC Genomics Workbench 8.0.3. The average nucleotide identity (ANI) was calculated using the online calculator at http://enve-omics.ce.gatech.edu/ani/index [13].

In addition, testing of an acute-stage plasma sample (collected on 03/02/2013) and two subsequent convalescent serum samples (collected on 03/14/2013 and 04/04/2013) taken from the patient was performed to detect lethal factor (LF), anti-protective antigen IgG, and anthrax lethal toxin neutralizing activity (TNA) immune response. All serological assays were performed as previously described [14, 15, 16].

Because the samples were collected in the course of clinical care and management, and sent to CDC for routine diagnostic purposes, informed consent was not obtained, and IRB approval was not sought. However, we received express written permission from the patient to publish the photograph presented in this report.

## Results

**Fig 2:**
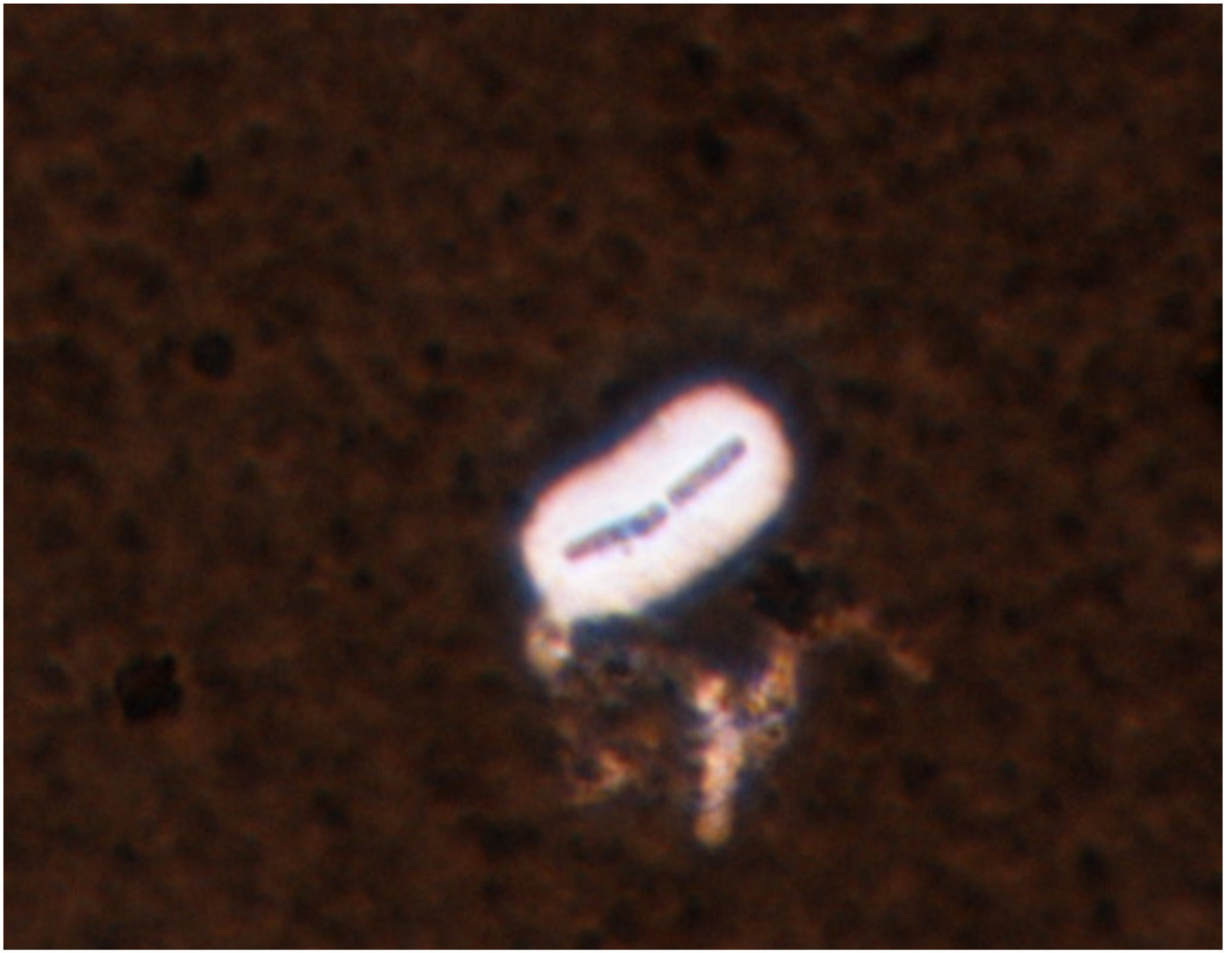
*B*. *cereus* BcFL2013 capsule. India ink stain of *B*. *cereus* BcFL2013 after overnight growth in defibrinated horse blood.

BcFL2013 was beta-hemolytic on sheep blood agar and resistant to gamma phage lysis (Table 1). The colony morphology was typical for *B*. *cereus*: large, umbonate (raised center), dark tan and granular. This isolate also produced a capsule (Table 1, Fig 2). Initial analysis of BcFL2013 by PCR revealed it was similar to G9241: positive for the *B*. *anthracis* pXO1-encoded toxin genes (*pagA*, *lef*, and *cya*) and negative for the pXO2-encoded capsule (*capA*) gene. However, unlike G9241, this isolate was negative by the pBC210 plasmid assay. Molecular subtyping showed the isolate had an identical MLST and *pagA* sequence type as G9241 (ST 78 and genotype 9, respectively).

**Table 1:** Summary of results for testing of *B*. *cereus* BcFL2013 compared to *B*. *cereus* G9241 and *B*. *anthracis* Ames.

Assay	*B*. *cereus* BcFL2013	*B*. *cereus* G9241	*B*. *anthracis* Ames
Susceptibility to gamma phage	Resistant	Resistant	Susceptible
Demonstration of capsule	Positive	Positive	Positive
MLST	78	78	1
*pagA* sequence type	9	9	6
pXO1 or homolog	Yes	Yes	Yes
pXO2 or homolog	No	No	Yes
pBC210 or homolog	Partial*	Yes	No

* Sequence with homology to approximately half of pBC210 was detected (108,352 bp).

We performed WGS to further characterize and determine the presence of virulence genes and plasmids in this isolate. Whole genome sequence Illumina reads for BcFL2013 were mapped to that of plasmid pBCXO1 of G9241, which indicated that a homolog of this plasmid is present, although with a ~ 2.5 kb deletion. Comparison of this homolog with pBCXO1 yielded an ANI of >99.98%. This included the anthrax toxin genes (*pagA*, *cya*, and *lef*) and hyaluronan capsule synthase genes (*hasA*CB) which were determined to be 100% identical to those found in pBCXO1. Mapping the Illumina reads of BcFL2013 to plasmid pBC210 of G9241 indicated a homolog of the plasmid was present but had only approximately half the sequence of pBC210 and did not include the *bpsXABCDEFGH* operon. The 108,352 kb of homologous pBC210 sequence had an ANI of 99.84% when compared to G9241.

Johnson et al. reported on the complete sequence of the third G9241 plasmid, originally named pBClin29 and noted as ~29-kb long, which they renamed pBFH_1 [17]. The complete pBFH_1 plasmid is 52,166 bp and encodes hypothetical phage proteins as previously described [2, 17]. Our sequencing of the BcFL2013 isolate revealed a 48-kb contig homologous to pBFH_1 (ANI of 98.74%). Analysis of the remaining reads (those not mapping to the chromosome or the three previously mentioned plasmids) did not indicate the presence of any additional plasmids.

The unmapped reads remaining after mapping to the three plasmids were used for a de novo assembly which yielded a putative chromosomal sequence ~ 5.14 MB. When compared to the sequence for the G9241 chromosome (CP009590.1), the putative BcFL2013 chromosomal sequence yielded an ANI of 99.98%. The shotgun sequence for BcFL2013 was deposited into DDBJ/EMBL/GenBank under accession no. JHQN01000000.

Serological testing of acute-stage plasma and two subsequent convalescent serum samples taken from the patient was performed to detect anthrax toxin lethal factor (LF), anti-protective antigen (PA) IgG, and anthrax lethal toxin neutralizing activity (TNA) immune response (Table 2). LF was detected in the acute sample (0.819 ng/mL) but not in the convalescent samples. These results were similar to levels observed for cutaneous anthrax cases described previously [18]. Anti-PA IgG was detected only in the convalescent samples (18.2 and 48.0 μg/mL, respectively). Similarly, anthrax lethal toxin neutralization activity was detected in the convalescent samples (ED50 = 156.5 and ED50 = 194.5, respectively) but was not tested for in the acute plasma sample.

**Table 2:** Results of serological testing of specimens from 2013 Florida patient with anthrax-like cutaneous lesion.

	Assay results		
Specimen and date collected	Lethal factor detection	Anti-PA ELISA	Toxin neutralization assay
Plasma (03/02/2013)	0.819 ng/mL	<LLOQ^b^	Not tested
Serum (03/14/2013)	<LOD^a^	18.2 μg/mL	156.5 (ED50)
Serum (04/04/2013)	<LOD	48.0 μg/mL	194.5 (ED50)

^a^LOD, limit of detection

^b^LLOQ, lower limit of quantification

## Discussion

To date, six *B*. *cereus* isolates, associated with human infections, have been described containing *B*. *anthracis* toxin genes: four isolates (G9241 from LA, 03BB87, 03BB102, Elc2) from metal workers in Texas and Louisiana with respiratory infections and two isolates (laboratory-acquired G9241 infection from Illinois and BcFL2013) resulting in cutaneous infections [2–5, 19]. In addition to these, the CDC received a *B*. *cereus* isolate harboring *B*. *anthracis* toxin genes (LA4726) from Louisiana in 2007 from a pneumonia patient who was also a metal worker (data not shown). Four of these isolates (03BB87, LA4726, G9241, and BcFL2013) had identical subtypes (ST 78) by MLST. The two remaining metal worker isolates (03BB102 and Elc2) were MLST subtypes ST 11 and ST 108, respectively. All seven metal worker isolates had sequence homology to *B*. *anthracis* toxin genes (*pagA*, *lef*, and *cya*) on pXO1. Only one isolate, 03BB102, had homology to the *B*. *anthracis* capsule genes (*cap* operon) on the pXO2 plasmid; however, it was not observed to produce a polyglutamate capsule like *B*. *anthracis* [3]. Phenotypically, all of the *B*. *cereus* isolates from the metal workers produced a capsule (either hyaluronic acid and/or exo-polysaccaride) although they differed in composition from the *B*. *anthracis* capsule. The BcFL2013 isolate described here has one of the capsule operons associated with G9241, *hasACB*. This would suggest that BcFL2013 produced, at very least, a hyaluronic acid capsule, but further studies would be required to determine the exact capsule composition.

In addition to these isolates, two *B*. *cereus* isolates have been isolated from non-human primates in Cote d’Ivoire and Cameroon (CI and CA) which harbor *B*. *anthracis* virulence genes and have been described as *B*. *cereus* biovar anthracis [6]. Unlike the strains from the metal workers, the *B*. *cereus* biovar anthracis isolates harbor both *B*. *anthracis* virulence plasmids (99–100% identity) [20]. Additionally, the CA and CI isolates have a non-functional *plcR* gene, a transcriptional regulator, due to an insertion at the 3’ end of the gene [6]. *B*. *anthracis* also has a non-functional *plcR* gene resulting from of a nonsense mutation in the gene [21]. Conversely, BcFL2013 has a predicted full length plcR protein with 100% identity to that of G9241.

Phenotypically, the CA and CI isolates differ from *B*. *anthracis* as they are motile, gamma-phage resistant, and, in some cases, penicillin-resistant and, unlike most *B*. *cereus*, are non-hemolytic on blood agar. [6]. The CA and CI isolates differ from the other *B*. *cereus* isolates in that they express both a hyaluronic acid and polyglutamate capsules [22]. By MLST, the CA and CI isolates, along with two metal worker isolates (03BB102 and Elc2), are more closely related to *B*. *anthracis* [3, 20]. It is of interest that, to date, the *B*. *cereus* biovar anthracis isolates have not been cultured from humans. Whether this is the result of virulence differences, decreased human exposure, or an artifact of limited diagnostic capacity in regions where these strains have been detected remains to be determined.

This is the first report of a *B*. *cereus* isolate harboring *B*. *anthracis* toxin genes associated with a naturally occurring cutaneous infection. However, as previously mentioned, there was a laboratory-acquired cutaneous infection associated with *B*. *cereus* G9241 in 2011 [19]. Unlike the previous cases associated with these types of *B*. *cereus* isolates, the Florida patient did not have any previous history of metal work. The patient had recent contact with apparently healthy horses but this is not a known risk factor for infection. Previous cases caused by these types of isolates occurred in Texas and Louisiana, neither of which were visited by this patient recently. It remains unknown how the Florida patient became infected.

In addition to being the first report of a naturally acquired *B*. *cereus* infection resembling cutaneous anthrax, this is the first report of demonstrated LF toxemia and a detectable humoral response to the toxin with *in vitro* toxin neutralization activity from an infection caused by a *B*. *cereus*. However, Brezillon et al. showed that the *B*. *cereus* biovar anthracis isolates expressed both *B*. *anthracis* toxin and capsule genes in animal models [22]. Previous reports of serious and fatal *B*. *cereus* infections did not include serologic testing of patients to determine if the toxin genes were expressed *in vivo* resulting in toxemia. These methods have been used successfully to detect toxin and antibody response in systemic and cutaneous anthrax cases [18, 23]. This case demonstrates that *B*. *anthracis* toxin genes are not only present but are also expressed *in vivo* and, in this case, associated with the clinical presentation resembling an anthrax eschar.
